# Dual-Toehold-Probe-Mediated Exonuclease-III-Assisted Signal Recycles Integrated with CHA for Detection of *mecA* Gene Using a Personal Glucose Meter in Skin and Soft Tissue Infection

**DOI:** 10.4014/jmb.2306.06037

**Published:** 2023-08-25

**Authors:** Jiaguang Su, Wenjun Zheng

**Affiliations:** Department of Dermatology, The First Affiliated Hospital of Guangxi Medical University, No. 6 Shuangyong Road, Nanning 530021, Guangxi Zhuang Autonomous Region, P.R. China

**Keywords:** *mecA* gene, *Staphylococcus aureus*, skin and soft tissue infections, exonuclease-III, personal glucose meter

## Abstract

*Staphylococcus aureus* integrated with *mecA* gene, which codes for penicillin-binding protein 2a, is resistant to all penicillins and other beta-lactam antibiotics, resulting in poor treatment expectations in skin and soft tissue infections. The development of a simple, sensitive and portable biosensor for *mecA* gene analysis in *S. aureus* is urgently needed. Herein, we propose a dual-toehold-probe (sensing probe)-mediated exonuclease-III (Exo-III)-assisted signal recycling for portable detection of the *mecA* gene in *S. aureus*. When the target *mecA* gene is present, it hybridizes with the sensing probe, initiating Exo III-assisted dual signal recycles, which in turn release numerous “3” sequences. The released “3” sequences initiate catalytic hairpin amplification, resulting in the fixation of a sucrase-labeled H2 probe on the surface of magnetic beads (MBs). After magnet-based enrichment of an MB-H1-H2-sucrase complex and removal of a liquid supernatant containing free sucrase, the complex is then used to catalyze sucrose to glucose, which can be quantitatively detected by a personal glucose meter. With a limit of detection of 4.36 fM for *mecA* gene, the developed strategy exhibits high sensitivity. In addition, good selectivity and anti-interference capability were also attained with this method, making it promising for antibiotic tolerance analysis at the point-of-care.

## Introduction

Infection with methicillin-resistant *Staphylococcus aureus* (MRSA) remains a serious public health issue worldwide [1-4]. MRSA acquires resistance to antibiotics through the integration of *mec* DNA, mainly the *mecA* gene, which codes for penicillin-binding protein 2a (PBP 2a) [5, 6]. Due to its resistance to all penicillins and other beta-lactam antibiotics, MRSA infections are associated with high rates of morbidity and mortality and can result in metastatic or complicated infections, such as skin and soft tissue infections (SSTI) or sepsis [7, 8]. Skin and soft tissue infections (SSTIs) cover a broad spectrum of diseases, account for a large proportion of infections requiring hospitalization, and are associated with high morbidity. Therefore, developing a biosensor capable of sensitively detecting the *mecA* gene in *S. aureus* is highly desired.

Microbiology culture is the conventional clinical technique for the detection of *S. aureus* [9-11]. Despite its widespread use, the classic culture method for detecting bacteria is tedious, labor-intensive, and limited by low sensitivity. Methicillin resistance can now be detected using a variety of technologies, including enzyme-linked immune-absorbent assay (ELISA) [12, 13], polymerase chain reaction (PCR) [14, 15], and antibiotic susceptibility testing (AST) [16]. These approaches offer increased sensitivity and specificity for microbiological detection, but they are time-consuming, complicated, and need extensive sample treatment, which limits their utility for point-of-care (POC) testing. Therefore, simple, sensitive, and low-cost diagnostic methods for drug resistance analysis of *S. aureus* are highly desired. Several recently developed methods, including electrochemistry [17], fluorescence [18], and gel electrophoresis [19] are available for use in MRSA investigation. However, these techniques require cumbersome equipment for signal readout, limiting their clinical applicability in resource-constrained settings.

As an alternative to conventional strategies, a portable glucose meter (PGM)-based approach has been gaining traction because of its low cost and low reagent and sample consumption [20, 21]. PGMs have been widely used in the investigation of biomarkers, small compounds, microorganisms, and cells. Due to the small sample volume, however, the sensitivity of the assay based on PGM is still below the intended levels. To surmount these obstacles, PGM-based strategies have been combined with various signal amplification techniques. For instance, Yuemeng Yang *et al*. reported a hybridization chain reaction-based approach for the detection of *S. aureus* with high sensitivity and a detection limit of approximately 2 CFU/ml [22]. However, the method was established with the PBP 2a, which is a lagging indicator. Using *mecA* gene as a detection target provided an earlier diagnosis of infection, but at the same time necessitated a method with significantly higher sensitivity.

**Fig. 1:**
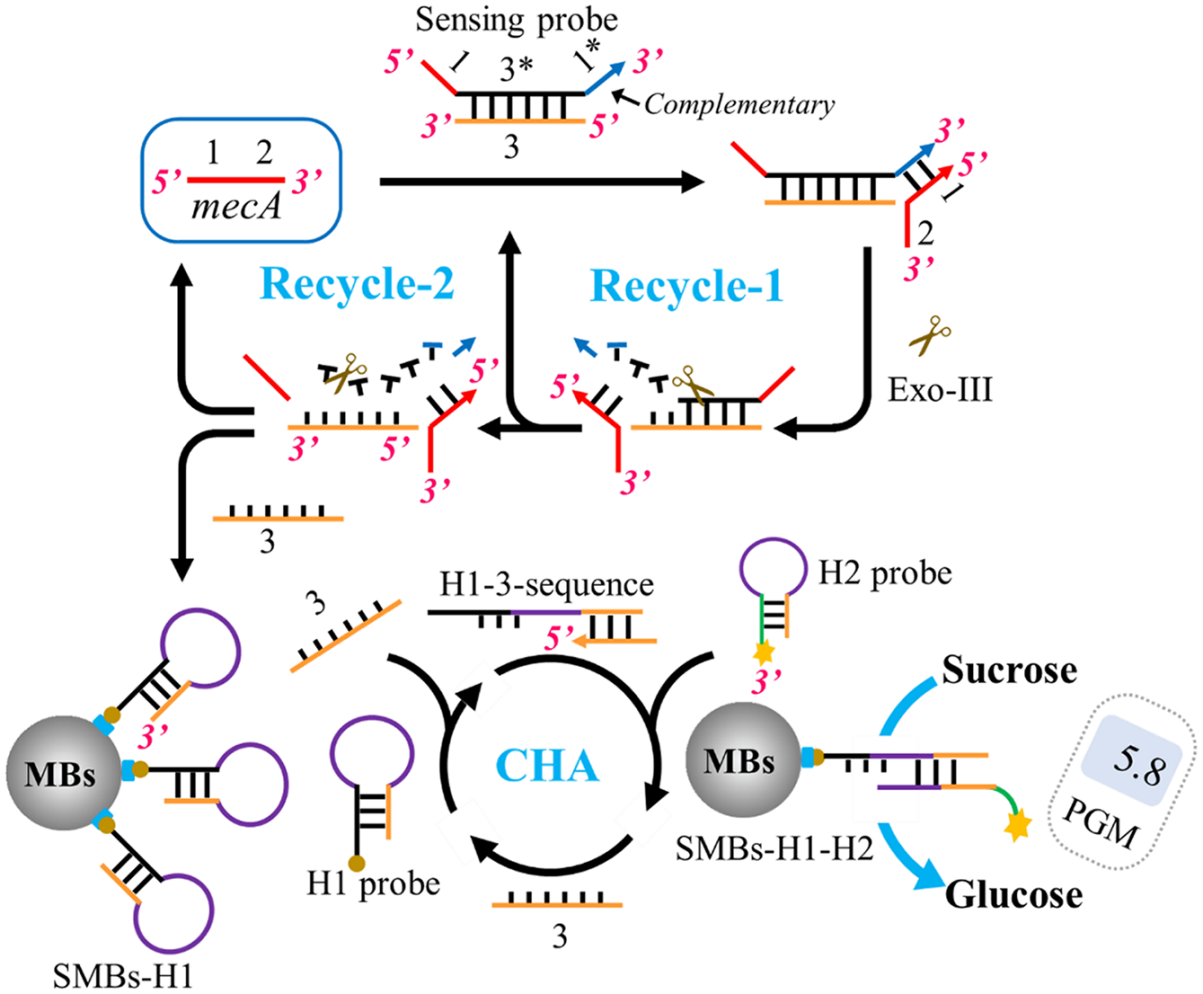
Schematic diagram of the dual-toehold-probe-mediated exonuclease-III- assisted signal recycles integrated with CHA for the detection of the *mecA* gene using personal glucose meter.

Herein, we developed a strategy integrating Exo-III assisted signal recycles with PGM to sensitively detect *mecA* gene in *S. aureus*. In this approach, a double-strand DNA probe (dsDNA probe) with dual toehold sequences, termed “sensing probe,” is constructed (Fig. 1). The sensing probe contains three functional portions, including the “1” portion, which is identical to the “1” portion in *mecA* gene, the “1*” that is complementary with “1” sequence, and “3” sequence to initiate subsequent signal amplification processes and to separate “1” portion and “1*” portion of the sensing probe via the formed dsDNA section. The “1” portion of *mecA* gene binds with the “1*” portion in the sensing probe and forms a blunt 3’ terminus. The Exo-III recognizes the blunt 3’ terminus of the sensing-probe-*mecA*-complex and digests “1” portion and the “3*” portion. When Exo-III cuts the “1” portion, the *mecA* gene is liberated to form a target recycle through binding with a next sensing probe (Recycle-1). When Exo-III digests “3*” sequence, “1” portion is released to attend the next signal cycle, and “3” sequence initiates a subsequent catalytic chain amplification (CHA)-based oxidation reaction. Before the CHA process, Exo-III is inactivated by heating the mixture to 70°C for 20 min. In this procedure, “3” sequence unfolds H1 probe to form H1-3-sequence complex, which will be replaced by the sucrose-labeled H2 probe, thereby forming CHA. As a result, the sucrose-labeled H2 probe is fixed on the surface of streptavidin magnetic beads (MBs) through hybridizing with H1 probe. After magnet-based enrichment of the MB-H1-H2-sucrase complex, the supernatant containing the free sucrose-labeled H2 probe is removed. The MB-H1-H2-sucrase complex is then used to catalyze sucrose to glucose, resulting in an enhanced PGM signal. In this way, the PGM signal can serve as a quantifiable and portable platform for detecting MRSA by directly reflecting changes in the concentration of the target *mecA* gene.

## Material and Methods

### Materials and Reagents

The nucleic acid sequences used in this research were synthesized and purified by the Shanghai Sangon Biotechnology Co., Ltd. (China). The details of the sequences are listed in Table S1. Exo-I, Exo-III, sucrase, and MBs were purchased from New England Biolabs (USA). Sucrose was provided by Beyotime Biotechnology Co., Ltd. (China). Tris-(hydroxymethyl) aminomethane (Tris) buffer solution (150 mM NaCl, 50 mM KCl, and 20 mM MgCl_2_, pH 7.5) and dimethyl sulfoxide (DMSO) were bought from Frontier Scientific Inc. (USA). The Yuwell GU200 PGM used for detecting glucose was obtained from Jiangsu Dive Kelit Biotechnology Co., Ltd. (China). All the obtained sequences were dissolved in 20 mM Tris-HCl buffer solution.

### Sensing Probe Assembly

To assemble the sensing probe, a 5 μl “3” sequence (5 mM) and a 5 Μl (5 mM) complementary sequence containing “1” portion, “1*” portion, and “3*” portion were mixed in a tube containing 10 μl Tris-buffer solution. The mixture was heated to 90°C for 10 min followed by cooling to 4°C at a speed of 1°C/min. Then, 5 μl Exo-I was added to the mixture for 10 min to digest the free ssDNA.

### Assay Procedure

Before detecting *mecA* gene, the H1 probe and H2 probe were annealed to hairpin structure. The annealed H1 probe was fixed on the surface of MBs through the interaction between biotin and streptavidin (experimental details included in electronic supporting information). For *mecA* detection, a 5 μl sensing probe was incubated with 5 μl *mecA* gene and 2 μl Exo-III (0.5 U/L) for 60 min at 37°C. The mixture was then heated to 70°C for 20 min to inactivate Exo-III. Subsequently, 5 μl of MB-H1 complex and 5 μl of H2 probe (5 mM) were introduced into the above solution and incubated at room temperature for 20 min. The mixture was put on a magnet and incubated for 15 min. After removing the liquid supernatant, the sediment was resuspended in 50 μl Tris-buffer solution. Then, 1 μl of a sucrose solution (0.5 M) was added to the mixture and incubated at 55°C for 30 min.

## Results and Discussion

### Construction of a Sensing Probe and Its Feasibility to Induce Exo-III-Based Signal Cycles

**Fig. 2:**
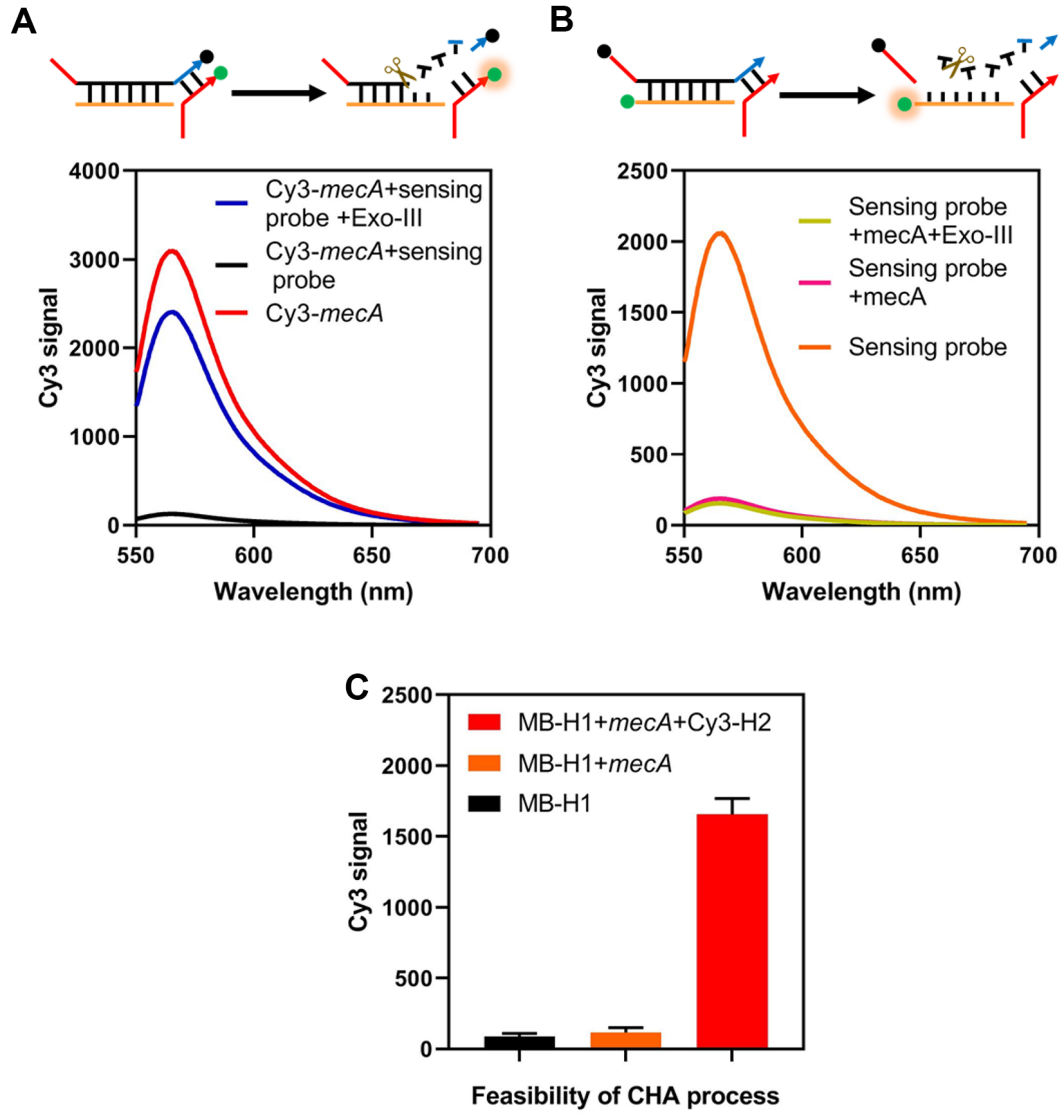
Feasibility of the Exo-III-assisted signal recycle and CHA process. (**A**) Cy3 signals of Cy3-labeled *mecA* gene with and without the existence of Exo-III. (**B**) Cy3 signals of Cy3-labeled “3” sequence in sensing probe with and without the existence of Exo-III. (**C**) Cy3 signals during the catalytic hairpin amplification (CHA) process.

To verify the feasibility of the sensing probe-mediated recycles, the fluorescence emission spectra were obtained under different assay conditions. To test the construction of the “Recycle-1”, the Cy3 moiety (Sulfo-Cyanine3) and BHQ were labeled on the terminus of the synthesized *mecA* sequence and “1*” portion in the sensing probe. The Cy3 signal was quenched by the BHQ moiety when *mecA* gene was mixed with the sensing probe, indicating the formation of a blunt 3’ terminal. Upon the addition of Exo-III, “1*” portion was digested and *mecA* gene was released. Consequently, Cy3 signal reappeared. As shown in Fig. 2A, the results demonstrated that Exo-III was activated and “Recycle-1” was constructed. Meanwhile, the formation of “Recycle-2” was investigated by labeling the Cy3 and BHQ moieties on the terminus of “1” portion and “3” portion. In the dsDNA state, the Cy3 signal is quenched. When Exo-III digested the “3*” section, “1” portion was released and the fluorescent signals recovered (Fig. 2B). These results clearly demonstrated that our DNA recycles can be utilized for the amplified detection of the target *mecA* gene. To verify the CHA process, Cy3 was substituted for the sucrase labeled at the end of the H2 probe. The Cy3 signals were recorded after magnet-based enrichment and removal of the liquid supernatant containing a free Cy3-labeled H2 probe. As depicted in Fig. 2C, in the absence of the “3” sequence and MB-H1 probe, signal levels were comparable to those of the control group. Only when all the necessary components were present was a significantly amplified Cy3 signal recorded, indicating that the CHA procedure was executed successfully.

### Optimization of Experimental Conditions

**Fig. 3:**
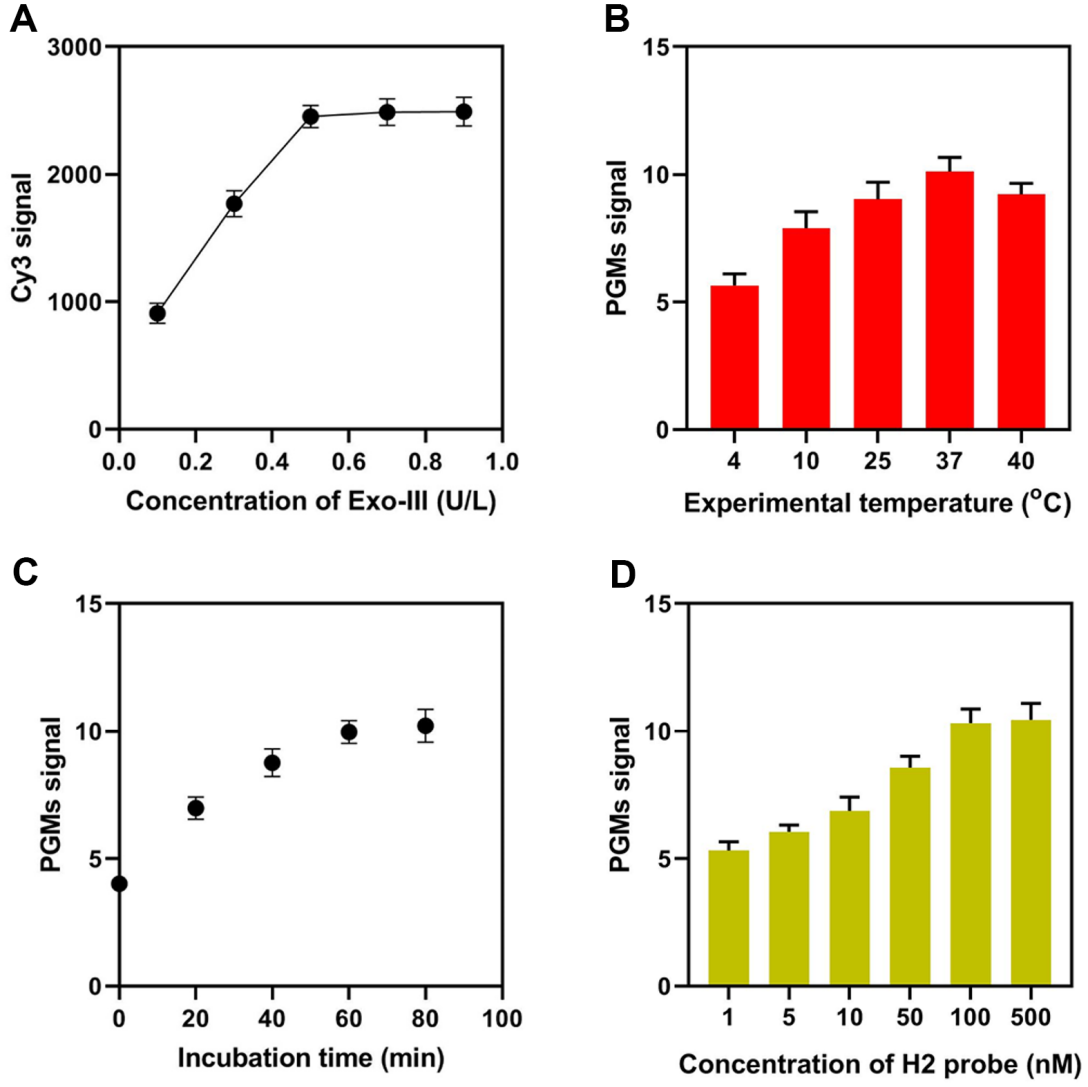
Optimization of experimental parameters. (**A**) Cy3 signals of Cy3-labeled *mecA* gene with different concentrations of Exo-III. (**B**) PGM signals of the approach with different experimental temperature, (**C**) incubation time, and (**D**) concentration of H2 probe.

Subsequently, the experimental conditions for the dual-toehold probe-mediated Exo-III-assisted signal amplification strategy for *mecA* detection were optimized further. Exo-III concentration is essential for mediating chain digestion and constructing signal recycles. Thus, the optimal Exo-III concentration was investigated. In this section, the terminals of the “1” portion of the sensing probe and the “3” sequence were labeled with Cy3 and BHQ, respectively. The recorded Cy3 signals increased as the concentration of Exo-III increased from 0.1 U/L to 0.5 U/L and reached equilibrium when the system was incubated with more Exo-III (Fig. 3A). Therefore, 0.5 U/L of Exo-III was used in the following experiments. Next, the optimal temperature for the experiment was determined. Under incubation at 4, 10, 25, 37, and 40°C, 10 nM of the *mecA* gene was detected, and PGM signals were captured. Fig. 3B demonstrated that when the sensing system was incubated at 37°C, the PGM signal was largely elevated, so this temperature was chosen for the subsequent investigations. For Exo-III-based signal amplification, reaction time was also a crucial factor. In addition, Fig. 3C showed that a 60-min reaction time yielded the greatest increase in PGM signal. Analytical performance could be affected by the concentration of the sucrase-modified H2 probe; as a result, it was optimized. The PGM signal increased with the sucrase-modified H2 probe concentration as shown in Fig. 3D. When the sucrase-modified H2 probe concentration was more than 100 nM, the PGM signal elevated gradually. Therefore, 100 nM was chosen as the optimal sucrase-modified H2 probe concentration.

### Analytical Performance of the Established Approach

**Fig. 4:**
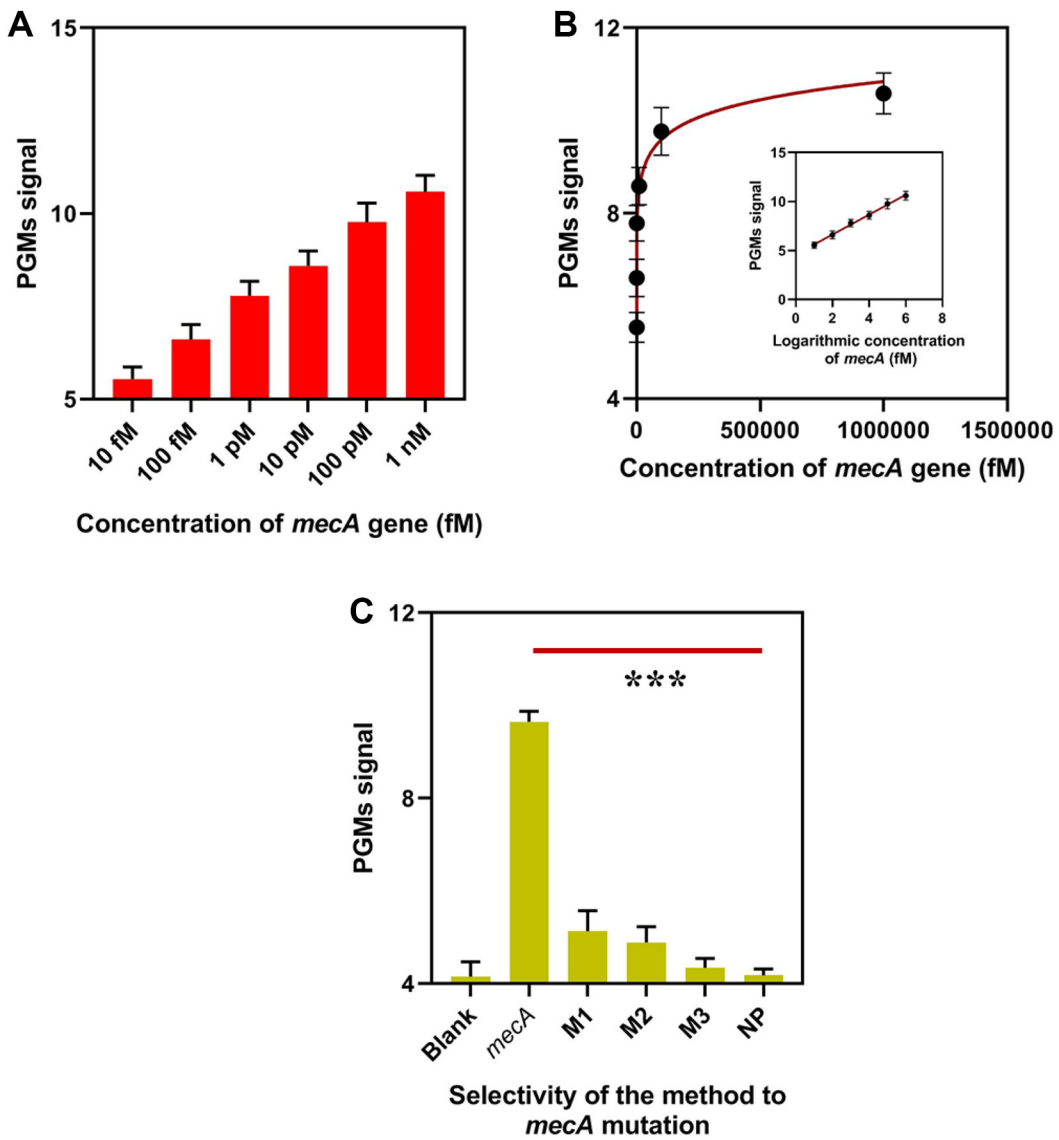
Analytical performance of the approach. (**A**) PGM signals of the approach when detecting different concentrations of *mecA* gene. (**B**) Correlation between the PGM signals and concentrations of *mecA* gene. (**C**) PGM signals of the approach when detecting *mecA* gene and mutations.

Quantitative detection of the target gene *mecA* was performed using the proposed approach in the established assay. As seen in Fig. 4A, the PGM signal steadily increased as the concentration of *mecA* gene increased from 10 fM to 1 nM. The PGM signal response exhibited a reasonable linear relationship with the logarithmic concentration of *mecA* gene, as depicted in Fig. 4B, and the linear equation was determined as *p*=1.015*lgC+4.593 (R^2^=0.9932) with a low limit of detection of 4.36 fM (S/N = 3). As interfering agents for the evaluation of the selectivity, 100 pM of *mecA* mutations, including M1 with a single base-pair mismatch, M2 with two base-pair mismatches, and M3 with three base-pair mismatches, were detected by the approach. As shown in Fig. 4C, the PGM signal of our proposed biosensor for 100 pM of the *mecA* gene was approximately 9.65. The PGM signals of the target with M1, M2, and M3 decreased to 5.13, 4.89, and 4.34, respectively. The PGM signal for NC and the blank sample is approximately 4.145. When there were more than three mutated bases in the target, the obtained PGM signal was almost identical to the baseline test, according to these findings.

### Real Sample Analysis

**Fig. 5:**
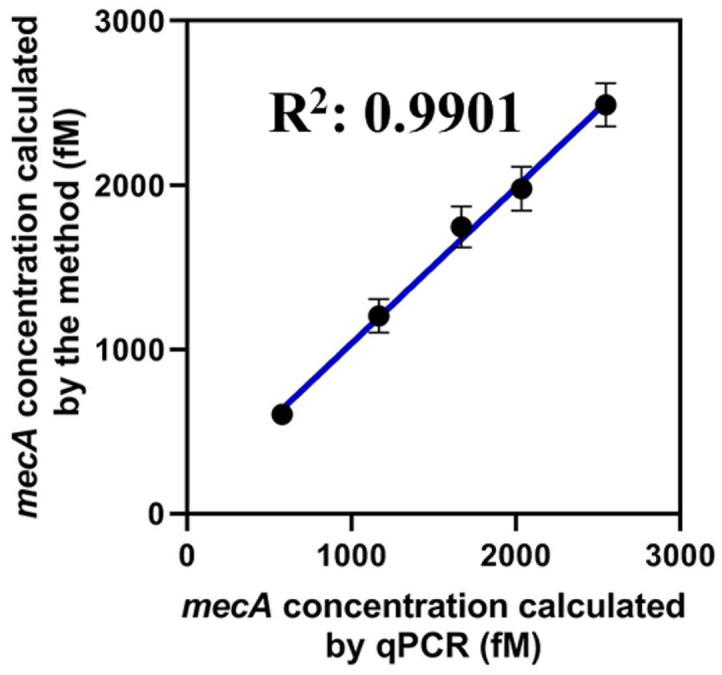
*mecA* concentration calculated by the proposed method and the qPCR method.

The method was applied to detect *mecA* gene from constructed clinical samples. The clinical samples were prepared by diluting different concentrations of *mecA* into commercial serum samples. The qPCR technique and the proposed method were utilized to determine the *mecA* levels in the samples. The results showed that the proposed method was accurate in detecting the target *mecA*, with no significant difference in the levels determined using qPCR (Fig. 5). These findings confirmed the accuracy of our manufactured biosensor in detecting *mecA* in clinical samples.

## Conclusion

In summary, we proposed here a portable approach by integrating dual-toehold-probe- mediated Exo-III-assisted signal recycles with a PGM-based signal readout for sensitive *mecA* gene detection. The developed PGM-based method completely eliminated the need for large, costly instruments, in contrast to other *mecA* detection methods that utilize various signal transducers like fluorescence signals, electrochemical signals, and colorimetric signals. The signal amplification provided by Exo-III-based signal recycling allowed for extremely sensitive detection, with a limit of detection of only 4.36 fM for *mecA*. In addition, the method has high selectivity and interference rejection. Compared with the traditional colony count method and other recently reported methods, the proposed approach possesses the advantages (Table S2) of i) direct *mecA* gene analysis; ii) Exo-III-assisted dual signal recycle coupling with CHA process endows the method with high sensitivity for *mecA* detection; iii) PGM is used in this approach for *mecA* gene analysis, which is portable. In the future, different biomarkers could be detected by simply replacing the sequence of the template strand, thereby broadening the applicability of the strategy and making it promising for miRNA detection at the point-of-care.

## Supplemental Materials

Supplementary data for this paper are available on-line only at http://jmb.or.kr.
