# Supplementary material for: Dual-Toehold-Probe-Mediated Exonuclease-III-Assisted Signal Recycles Integrated with CHA for Detection of mecA Gene Using a Personal Glucose Meter in Skin and Soft Tissue Infection
Source: J Microbiol Biotechnol. 2023 Aug 25;33(12):1692–7. doi: 10.4014/jmb.2306.06037 (PMC10772588; doi:10.4014/jmb.2306.06037)
Supplement: Supplementary file 1 [file jmb-33-12-1692-supple.pdf]

J. Microbiol. Biotechnol.
https://doi.org/10.4014/jmb.2306.06037

Electronic supporting information

Dual-toehold-probe mediated exonuclease-III assisted signal recycles integrating with CHA for the detection of the *mecA* gene using a personal glucose meter

Table S1. The nucleic acid sequences used in the experiment.

Title	Sequence
Biotin-H1	Biotin-AAA AAC AAG GAA CAA CAT TGC CAG TCA CGA CAT CAT GGC AAT GTT GTT CCT TGA GGA AG
H2	Sucrase-AAA AAG TCA CGA CAT CAT GGC AAC AAG GAA CAA CAT TGC CAT GAT GTC GTG ACT GGC AA
“3”	CTT CCT CAA GGA ACA ACA TTG CCA
mecA	ATT GGG ATC ATA GCG TCA TT
Complementary	ATT GGG ATC ATA GCG TCA TTC AAT GTT GTT CCT TGA GGA ATG AAC GCT ATG ATC CCA AT

Supplementary experimental section

Preparation of H1 probe-Modified MBs. A total of 20 μL of MB-SA (10 mg/mL) was mixed with 40 μL of H1 probe for 1 h. The synthesized H1 probe-modified MBs were washed with PBST three times and then dispersed in 20 μL of PBS for further use.

PCR method for *mecA* analysis:

The synthesized *mecA* sequences in the constructed clinical samples were subjected to real-time PCR. Briefly, a 25-μL reaction mixture containing 12.5 μL of 2× TaqMan universal PCR master mix, 2.5 μL of 10 pM/μL forward primer (primer F mecA), 2.5 μL of 10 pmol/μL of reverse primer (primer R mecA), target mecA gene, and nuclease free-water was prepared and subjected to real-time PCR with an ABI Prism 7900 sequence detector (Applied Biosystems, Foster City, CA). The amplification conditions were 50°C for 2 min and 95°C for 10 min, followed by 50 cycles of 95°C for 15 s and 60°C for 1 min.

Table S2. A brief comparison of the proposed method with former ones.

**Title**	**Target**	**Mechanism**	**LOD**	**Signal mode**	**Disadvantages**	**Ref**
The method	*mecA* gene	Exo-III assisted signal cycles; CHA	4.36 fM	PGMs signal	Requiring inactivation of Exo-III.	
Electrophoretic microchip	*mecA* gene	Isothermal strand-displacement polymerase reaction	12.3 pM	Fluorescent signal	High cost; Low sensitivity.	[1]
DNA circuit	*mecA* gene	Exo-III and DNAzyme-mediated signal amplification	0.5 fM.	Fluorescent signal	Requiring cumbersome equipment to record fluorescent signals.	[2]
G-quadruplex based method	*mecA* gene	Exo-III assisted cascade signal amplification strategy	2.4 fM	Fluorescent signal	Requiring cumbersome equipment to record fluorescent signals.	[3]
Aptamer based method	SA	Hybridization chain reaction strategy	2 CFU/mL	PGMs signal	Surface protein analysis; cannot analysis drug tolerance of SA.	[4]
Colony count method	Colony analysis			Bacterial colony count	High labor intensity; time-consuming.	

SA, *Staphylococcus aureus*; Exo-III, exonuclease-III; PGMs, personal glucose meters.

References

1. Lu Y, Luo F, Li Z, Dai G, Chu Z, Zhang J, *et al.* 2021. Ultrasensitive microchip electrophoretic detection of the mecA gene in methicillin-resistant Staphylococcus aureus (MRSA) based on isothermal strand-displacement polymerase reaction. *Talanta.* **222:** 121686.
2. Pan J, Bao D, Bao E, Chen J. 2021. A hairpin probe-mediated DNA circuit for the detection of the mecA gene of Staphylococcus aureus based on exonuclease III and DNAzyme-mediated signal amplification. *Analyst* **146:** 3673-3678.
3. Li Q, Zhou D, Pan J, Liu Z, Chen J. 2018. Ultrasensitive and simple fluorescence biosensor for detection of the mecA gene of Staphylococcus aureus by using an exonuclease III-assisted cascade signal amplification strategy. *Analyst* **143:** 5670-5675.
4. Yang Y, Wu T, Xu LP, Zhang X. 2021. Portable detection of Staphylococcus aureus using personal glucose meter based on hybridization chain reaction strategy. *Talanta* **226:** 122132.
